# 4D-printed hybrids with localized shape memory behaviour: Implementation in a functionally graded structure

**DOI:** 10.1038/s41598-019-55298-1

**Published:** 2019-12-10

**Authors:** Yu-Chen Sun, Yimei Wan, Ryan Nam, Marco Chu, Hani E. Naguib

**Affiliations:** 1 Department of Mechanical and Industrial Engineering, University of Toronto, Toronto, Canada; 2 Department of Materials Science and Engineering, University of Toronto, Toronto, Canada; 3 Institute of Biomaterials and Biomedical Engineering, University of Toronto, Toronto, Canada

**Keywords:** Polymers, Mechanical engineering

## Abstract

4D-printed materials are an emerging field of research because the physical structure of these novel materials respond to environmental changes. 3D printing techniques have been employed to print a base material with shape memory properties. Geometrical deformations can be observed once an external stimulus triggers the shape memory effect (SME) integrated into the material. The plasticizing effect is a well-known phenomenon where the microscopic polymer chain movements have been altered and reflected in different shape memory behaviour. It has been suggested that a 4D material with localized actuation behaviour can be fabricated by utilizing functionally graded layers made from different degrees of plasticizing. This study demonstrated that a novel 4D material can be fabricated from material extraction continuous printing technique with different loadings of poly(ethylene glycol) (PEG) plasticize, achieving localized thermal recovery. The results indicate that a plasticized functional layer is an effective technique for creating next generation 4D materials.

## Introduction

Additive manufacturing, also known as three-dimensional (3D) printing, is an emerging fabrication technique for creating complex 3D objects by utilizing a layer-by-layer deposition method^1–3^. In the past decade, a number of different 3D printing technologies have been proposed and developed in both academia and industry. For example, fused deposition modelling (FDM) extrudes molten polymer^4,5^, binder jetting deposits adhesive agents onto powder materials^6,7^, and stereolithography (SLA) uses the photopolymerization technique to cure and solidify liquid polymer resin^8–10^. Due to the variety of available printing technologies, additive manufacturing applications include rapid physical prototype fabrications^11–13^, biological and healthcare products^14,15^, functional electronic device fabrications^16,17^, and honeycomb structures^18^.

Advances in novel material development have increased the number of materials that can employ additive manufacturing, particularly 3D-printed composites and customized materials^19,20^. These application-driven composite materials may have superior mechanical^19^, thermal^21^, and electrical properties^22^ and can be fabricated into complex geometries. Smart materials in 3D printing also draw significant attention for their ability to respond to external stimuli^23–25^. Temperature^26,27^, water^28,29^, and light^30,31^ are potential stimuli that can cause shape memory polymers (SMPs) to recover to a pre-defined shape after plastic deformation. Because the geometry in smart material-based 3D-printed components can change over time, a new term, “4D printing,” was established^11,23–25,32^. Compared to traditional 3D-printed parts, the additional dimension in the time domain allow for the components to undergo dramatic changes in shape when subjected to an external stimulus. Similar to SMPs, stimuli that induce time-dependent behaviours are usually temperature changes^33–35^ or water exposure^11,36,37^.

**Figure 1:**
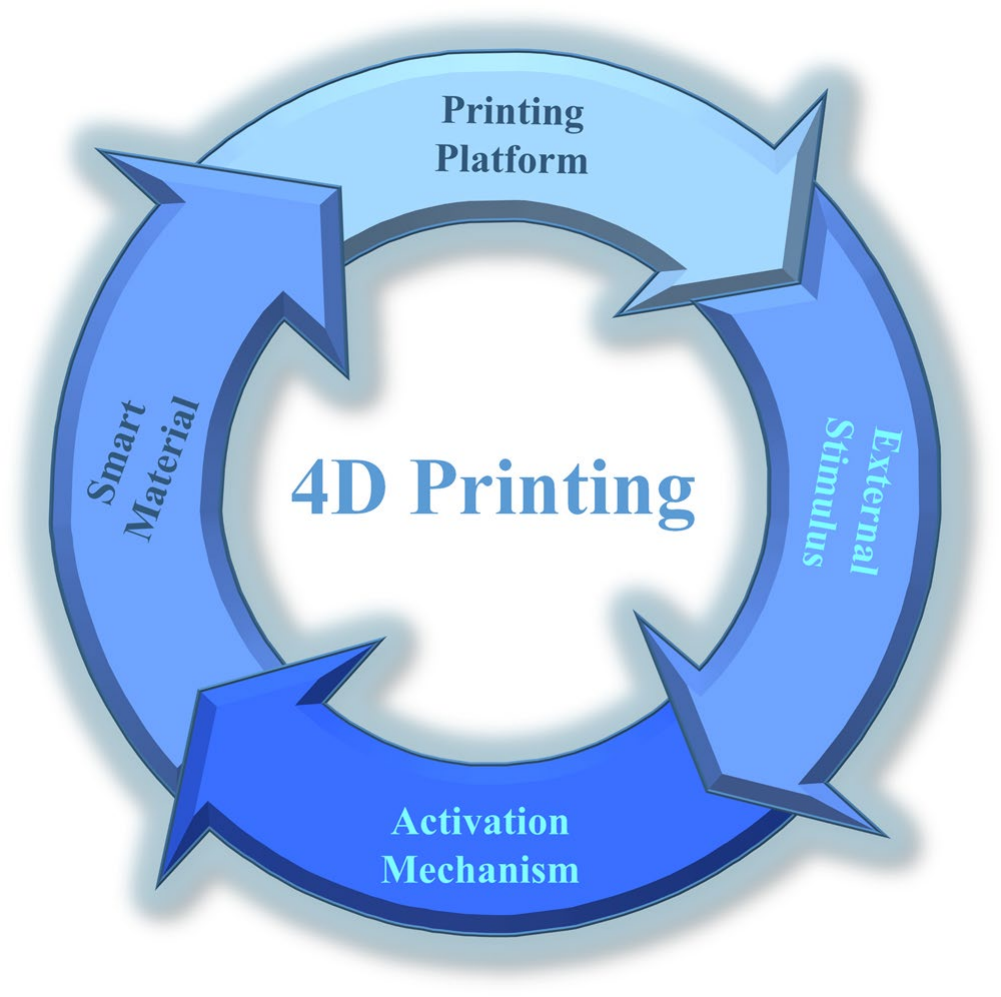
Basic building blocks for 4D printing development.

Four major building blocks for 4D printing development have been identified and are shown in Fig. 1. These include (1) a proper 3D printing facility/platform with a pre-determined printing path, (2) a suitable smart or stimulus-responsive material, (3) an external stimulus for activation, and (4) a known activation/interaction mechanism, such as a shape memory effect (SME)^24^. These building blocks are interconnected such that they function together to create an effective 4D material. The type of printer platform depends heavily on the materials used, and these materials may only respond to certain stimuli. In addition, the macroscopic shape memory behaviour depends not only on the material properties, but also on the fabrication process parameters, such as printing direction and composite layout. As a result, all four components must be carefully considered during 4D printing development.

4D-printed components can be created through a number of different methods. The polyjet printer system dispenses photocurable SMP using a bottom-up method that cures each layer separately^11,32,38^. This is similar to the direct-writing methods proposed by Lewis *et al*.^36,39,40^. In comparison, SLA printers directly cure liquid polymer resin and can also be used in 4D printing applications^16,41^.

Conventionally, 4D printing involves printing from mono material resin. In 2013, Ge *et al*. provided one of the earliest reports of the fabrication of 4D-printed active composites (PACs) by utilizing fiber/matrix configurations^38^. The group deposited shape memory polymer fibers within a UV-curable elastomer matrix. The fibers exhibited SME between 15 °C to 60 °C, and the matrix underwent a glass transition (T_g_) below –5 °C. Thus, the thermomechanical properties of the elastomer do not influence the shape-shifting behaviour of the fibers during the recovery process. When changing the PAC into a temporary shape, mechanical deformation must be applied at a high temperature after printing. Reheating the deformed PAC can change the PAC back to its printed shape. In addition to the conventional shape memory behaviour, the group also demonstrated that anisotropy behaviour can be achieved through fiber alignment using a laminated structure.

Despite the widespread availability of FDM printers on the market, there are relatively few studies that have focused on FDM methods for creating 4D materials^42^. Recently, Ly *et al*. demonstrated that 4D objects can be obtained from directly printing SMP material^43^. It is possible to achieve electroactive ability in SMP materials by blending multiwalled carbon nanotube (MWCNT) into the thermoplastic system. Similarly, Hu *et al*. established the self-bending ability of FDM-printed SMP and constructed a constitutive model for its recovery behaviour^44^. Bodaghi *et al*. used polyurethane-based SMP filaments to create FDM-printed 4D SMP metamaterial^45,46^. The team also attempted to create a functionally graded (FG) layer by varying the printing conditions^46^.

Recent 4D printing studies have shown the capabilities of an FDM-printed sample; nevertheless, the SMP materials used in these studies were directly purchased from the market and were not custom-formulated. It is proposed that optimized 4D performance and recovery can be achieved by redesigning the material properties from microscale crystalline/amorphous structures to macroscale 3D-printed layers. For example, the term FG layer used in^46^ provides interesting insight into next-generation 4D materials despite the lack of material property characterization tests on the different functional layers. In the literature, FG materials are defined as composites with gradual property variation throughout the object^47,48^. Due to the anisotropic design, properties such as specific crack propagation^49^ and superior electrical conductivity can be achieved^50^. The FG material concept can also be implemented in SMP composites. For instance, DiOrio *et al*. created a thermoset SMP with multiple layers, and each had a different transitional temperature^51^. Lu *et al*. created different FG layers of carbon nanofiber (CNF) and boron nitride (BN) nanopaper onto a SMP to achieve electroactive ability^52^.

Printing technologies further simplify the fabrication of functionally graded material (FGM). It is possible to achieve multi-shape or revisable SMP through 4D printing due to the designated differences in each functional layer^33,53^. Although these 3D-printed FGM have shown interesting properties and performance, the functional layers are still constructed from a single base material rather than composites. For instance^46^, simply varied printing parameters, such as the speed and temperature of each FG layer. A plasticizer can be used alongside the base material to further develop 4D-printable FGM. Polylactic acid (PLA) is one of the most commonly used materials in FDM due to its low cost and high stiffness^54^. Furthermore, the plasticizing effect of PLA and the thermal/rheological/mechanical properties that are induced as a result are well-documented^55–59^. Moreover, several groups have reported the SME of 3D-printed PLA^60–63^. In our previous study, we also demonstrated that the plasticized SMP may have superior recovery properties^64^. This study showed that it is possible to create a 4D FDM-printed multi-shape memory composite with localized actuation properties by implementing the plasticizing effect. A tri-layer FG 4D composite with increasing plasticizer content across the gradient was fabricated. PLA was first plasticized with different amounts of poly(ethylene glycol) (PEG) to alter the mechanical and thermal properties at the microscopic level. Due to changes in the polymeric chain movements, the macroscopic SME could be controlled by 4D printing different FG layers to achieve multi-shape memory and localized actuation properties.

## Results and Discussion

### Plasticizer-induced microscopic morphological changes

**Figure 2:**
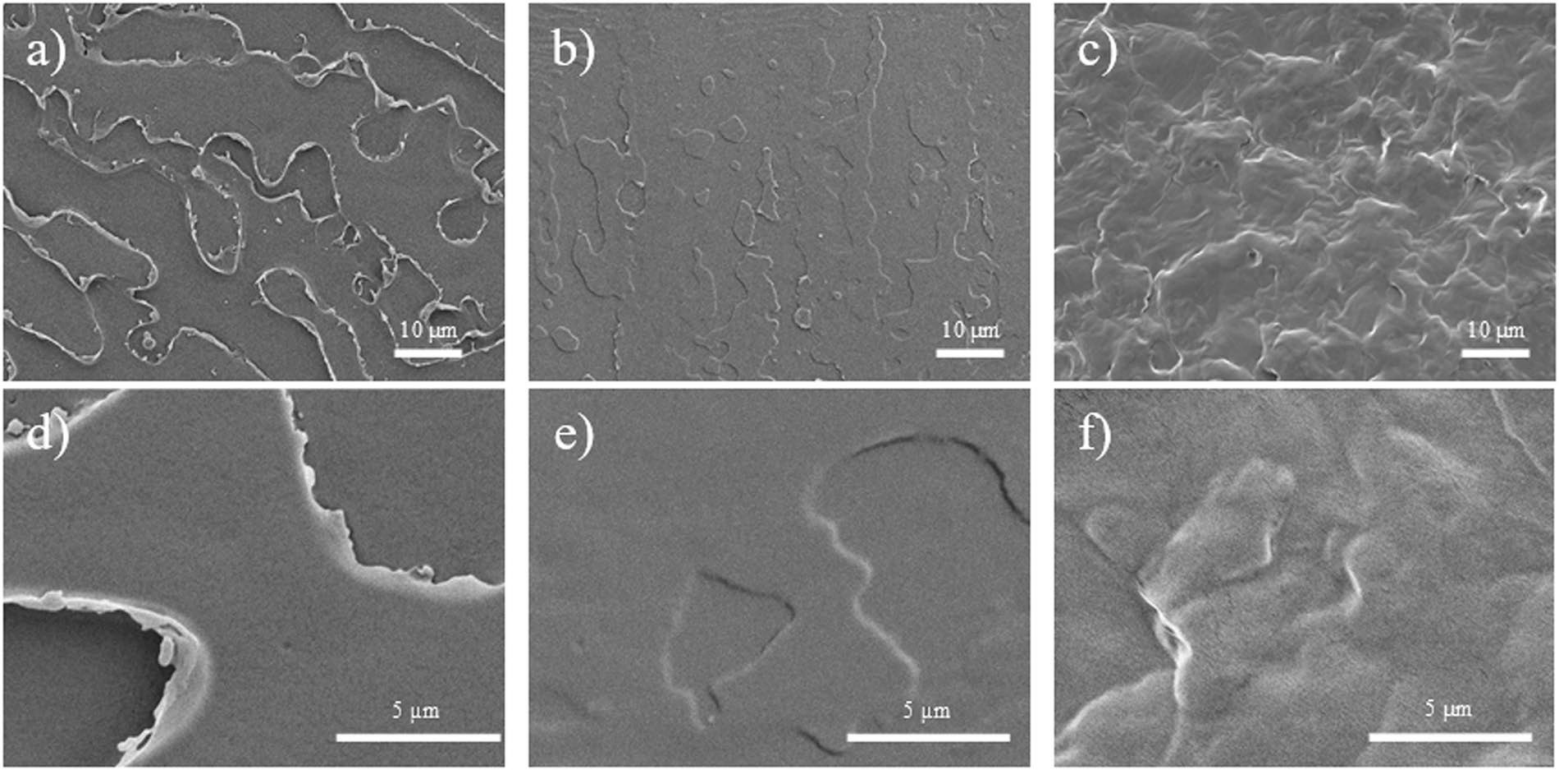
Fracture surfaces of (**a**,**d**) PLA, (**b**,**e**) 10PEG, and (**c**,**f**) 30PEG.

**Figure 3:**
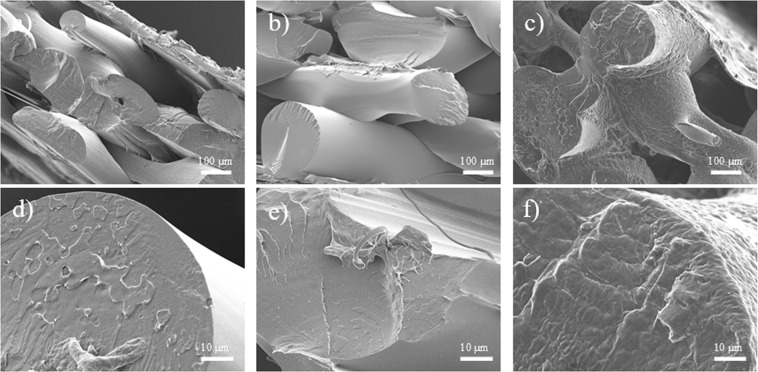
3D printed fracture surfaces of (**a**,**d**) PLA, (**b**,**e**) 10PEG, and (**c**,**f**) 30PEG.

It was expected that the microstructure of the PLA matrix would be altered due to the presence of PEG plasticizer. In Figs 2a,d, it can be inferred that the material has a brittle fracture behaviour because of the sharp surface defects. With an increasing PEG content, the sharp edges begin to smoothen as the mechanical ductility of the material improves^55,56^. Increasing surface roughness and fibril structures can also be observed, which is similar to the results reported in literature^55^. To further verify the effect of FDM printing on the material, the 3D-printed components were subjected to liquid nitrogen fracture, and the morphologies are shown in Fig. 3. All three materials were printed successfully as clear extruded polymer filament layers can be observed. The cross-sections of the extruded layer (Fig. 3d–f) also show similar fracture characteristics when compared to those of the pre-extruded composites (Fig. 2). Brittle fracture edges are suppressed and replaced by fibril-like structures due to the induced plasticizing effect of PEG. In addition to the cross-sectional surface, the ductile behaviour can also be observed on the exterior surface layer under high PEG content as shown in Fig. 3c,f.

### Chemical composition verification

**Figure 4:**
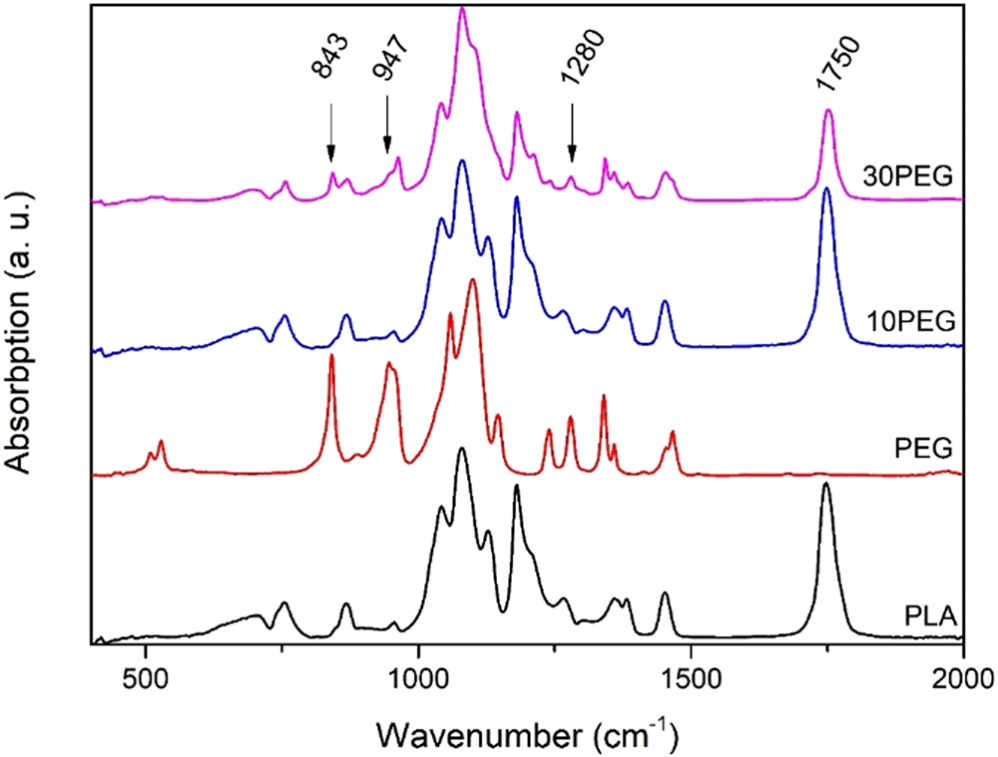
FTIR absorption spectra of PLA/PEG composites.

The FTIR spectra of PLA with different PEG contents are shown in Fig. 4. For PLA, the C=O group was represented by a characteristic peak at 1750 cm^−1^ ^65,66^, and was only present in samples containing PLA. Three different peaks characteristic of PEG were at 843 cm^−1^, 947 cm^−1^, and 1280 cm^−1^ ^67,68^. These peaks were relatively weak when PEG content was low (10 wt%) and could only be identified when PEG content was increased to 30 wt%.

### Thermal behaviour of plasticized PLA composites

**Figure 5:**
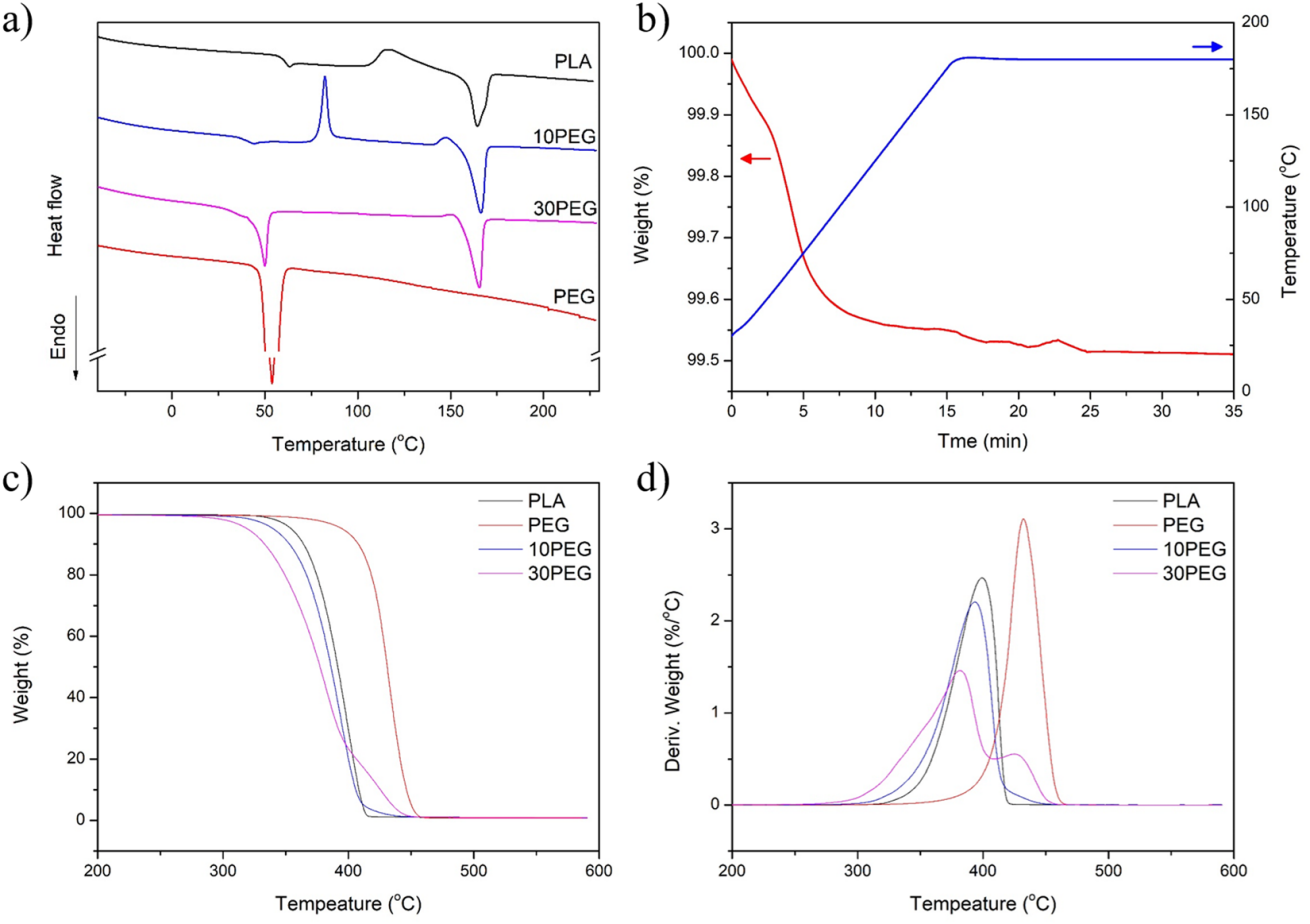
(**a**) DSC curves of PLA/PEG composites, (**b**) thermal stability of PEG at 180 °C, (**c**) thermal degradation, and (**d**) the derivative of thermal degradation curves.

Differential scanning calorimetry (DSC) experiments and thermal thermogravimetric analyses (TGA) were conducted to analyze the thermal behavior (Fig. 5). Table 1 summarizes the glass transition (T_g_), cold crystallization (T_cc_), melting temperature (T_m_), cold crystallization enthalpy (ΔH_cc_), melting enthalpy (ΔH_m_), and percent crystallinity (Xc). Table 2 summarizes the initial degradation (T^i^), primary maximum weight loss slope (T^i^_max, 1_), and secondary maximum weight loss slope (T^i^_max, 2_) from the TGA/DTG curves. The percent crystallinity was calculated by subtracting the ΔH_cc_ from the ΔH_m_ then dividing by the ΔH_m_ of a perfect crystal (197 J/g for PEG and 93.6J/g for PLA)^69,70^.

**Table 1:** Summary of thermal properties of PLA/PEG composites.

	T_g_ (°C)	T_cc_ (°C)	T_m_ (°C)	ΔH_cc_ (J/g)	ΔH_m_ (J/g)	Xc (%)
PLA	61.32	115.39	166.78	16.09	24.29	8.70
PEG	N/A	N/A	53.91	N/A	177.30	90.00
10PEG	41.54	82.42	166.44	18.12	34.85	17.87
30PEG	N/A	N/A	165.61	N/A	36.06	38.53

**Table 2:** Summary of initial degradation temperatures and maximum weight loss.

	PLA	PEG	10PEG	30PEG
T^i^ (°C)	334.60	361.67	313.76	281.34
T^i^_max, 1_ (°C)	399.35	432.25	393.54	381.73
T^i^_max, 2_ (°C)	N/A	N/A	N/A	424.92

One of the most noticeable changes caused by the plasticizing effect was the decrease in Tg (Fig. 5a and Table 1). Because the PEG molecules are much smaller than the PLA polymeric chains, the plasticizer can easily penetrate the PLA matrix and enhance polymeric chain movement, thus lowering the T_g_^71,72^. At 30 wt% PEG, the T_g_ of PLA could no longer be observed since the melting behaviour of PEG was dominant in the same temperature region^71^. The presence of PEG also enhanced PLA formation and was observed as an increase in melt enthalpy. It is possible that the plasticizer molecules lowered the interfacial surface energy and promoted PLA crystal nucleation^71,73^.

To verify the observed thermal stability and processability, a heat-and-hold experiment was conducted on PEG at 180 °C (Fig. 5b). The material was thermally stable for a period of 35 min as the overall weight lost was less than 0.5%. Based on the TGA curves (Fig. 5c), no material residue was left after thermal degradation above 500 °C as complete decomposition behaviour was observed. Both T^i^ and T^i^_max_ were lowered once the plasticizer was incorporated into the PLA matrix due to the enhanced PLA/PEG interaction. With 30 wt% PEG, a two-step degradation behaviour resulted from the high PEG content. Figure 5d shows that the peaks for the T^i^_max 2_ of the 30PEG curve closely matched the DTG curve of pure PEG. This result suggests that PEG crystalline formation may have occurred within the composites, and a continuous phase was no longer present in the PLA/PEG composites.

### Viscoelastic and rheological characterizations

**Figure 6:**
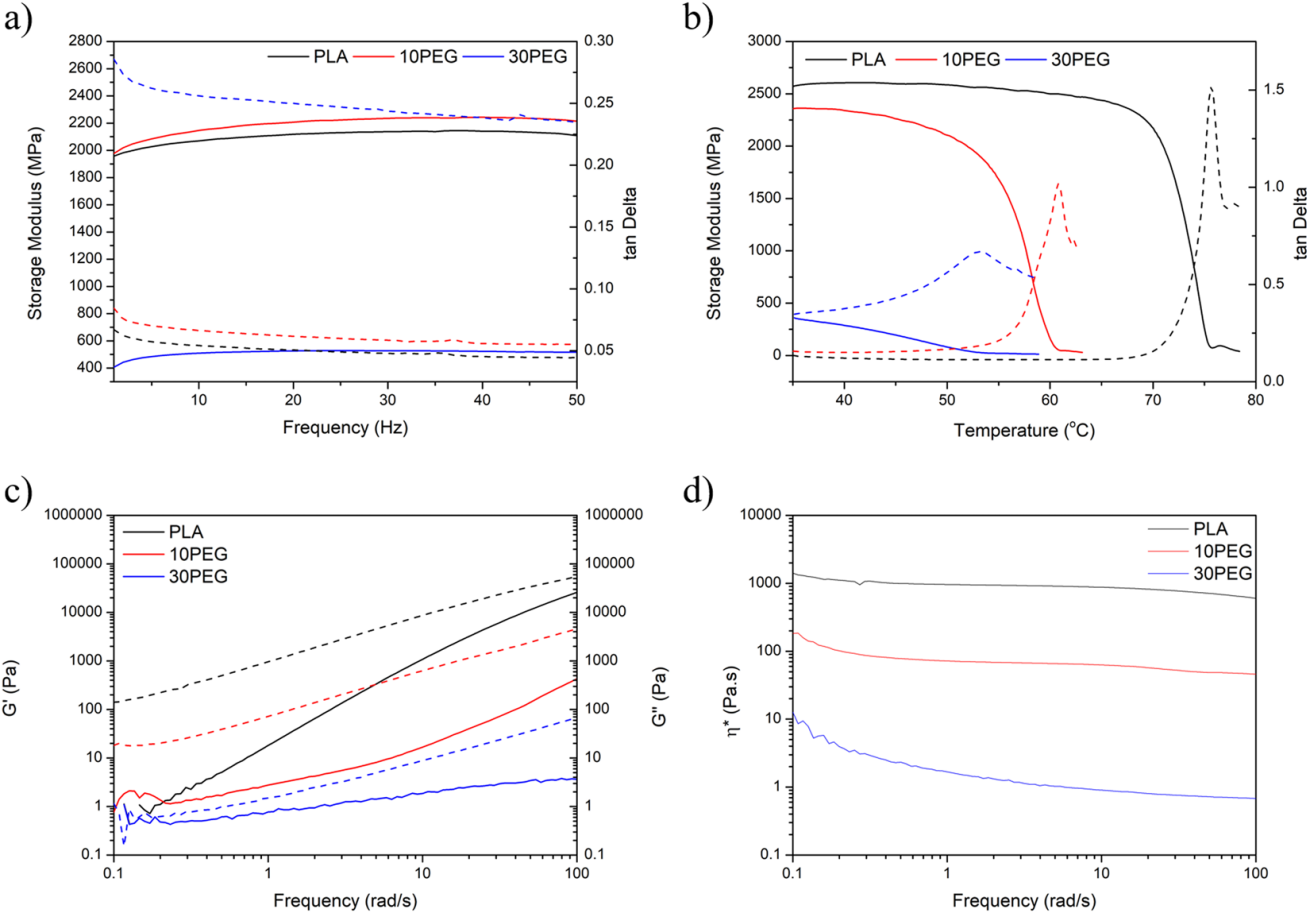
(**a**) Dynamic frequency sweep, (**b**) temperature sweep from dynamic mechanical testing; (**c**) storage/loss moduli and (**d**) viscosity measurement.

To characterize the viscoelastic behaviour of the composite, dynamic mechanical tests were conducted under two conditions: a room temperature frequency sweep (Fig. 6a) and a single-frequency temperature sweep (Fig. 6b). Both the storage modulus and tan Delta values were affected by the frequency at room temperature. A slight increase in the storage modulus of the 10PEG sample may contribute to the higher crystallinity as shown in the thermal analysis. With 30% PEG, the storage modulus dropped significantly because of the plasticizing effect in the PLA matrix. In Fig. 6b, the storage modulus displayed temperature-dependent behaviour. With increasing PEG content, the tan Delta peak shifted to a lower temperature, which was represented as a decrease in T_g_^71^.

The plasticizer effect was also monitored in rheological studies (Fig. 6c–d). At a high temperature (190 °C), the storage modulus of PLA increased with frequency. The increase in the amount of PEG present in the matrix caused the slope to decline, which suggests that the viscoelastic behaviour deviated from a typical homogeneous polymer. The data suggest that the plasticizing effect resulted in a significant reduction in the viscosity because the polymer chain movements increased as a result of the presence of PEG (Fig. 6d)^64,72^.

### Temperature dependant mechanical properties

**Figure 7:**
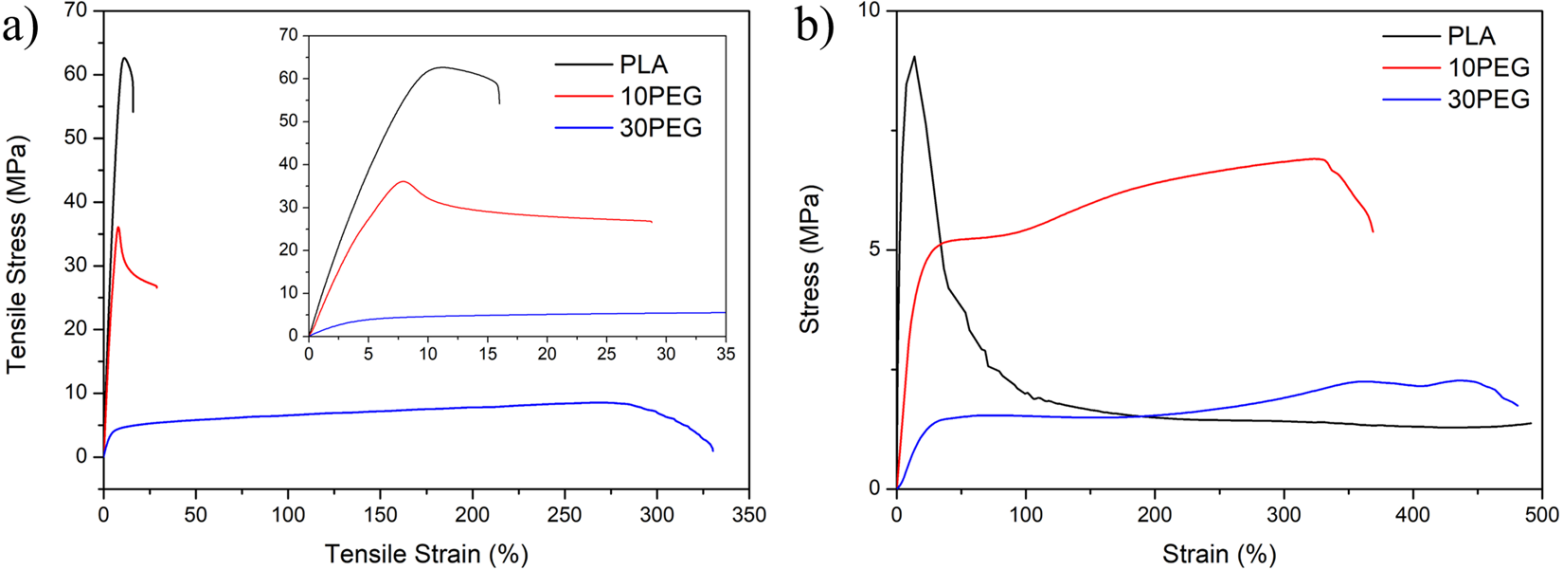
Stress strain curves of PLA/PEG composites at (**a**) room temperature and (**b**) 70 °C.

The stress-strain behaviour of the fabricated PLA and plasticized composite is shown in Fig. 7. The mechanical properties, including the elastic modulus, ultimate yield strength, and elongation at break, are also summarized in Table 3 and Table 4. Without any modifications, PLA exhibited a brittle behaviour with extremely low elongation at break as reported in literature^60,74^. The increasing plasticizing content caused a decrease in tensile strength and an increase in elongation at break^74,75^. Pillin *et al*. reported that up to 200% strain can be reached when 20 wt% of PEG with 1000 molecular weight was blended with PLA. The further addition of PEG may result in a decrease in the tensile strain because of an increase in the immiscibility^75^. In this study, the 30 wt% PEG/PLA blend reached a strain of over 250% before tearing under room temperature conditions. Kokturk *et al*. provided one of the earliest reports of high temperature and stretching-induced crystallization behaviour of PLA film^76^. The group demonstrated that PLA can hold a stretch ratio of up to 500% at 70 °C as the stretching was performed at temperatures above the T_g_ of PLA. As shown in Fig. 7b, all three composites sustained a striation ratio of at least 300% and, therefore, were suitable to be tested for shape memory properties.

**Table 3:** Mechanical properties under room temperature testing condition.

	PLA	10PEG	30PEG
Elastic Modulus (MPa)	844.84 ± 26.80	652.91 ± 66.21	119.92 ± 16.09
Ultimate Yield Strength (MPa)	54.35 ± 4.06	36.66 ± 6.96	8.25 ± 0.43

**Table 4:** Mechanical properties under 70 °C testing condition.

	PLA	10PEG	30PEG
Elastic Modulus (MPa)	248.36 ± 11.42	33.47 ± 6.44	3.17 ± 0.42
Ultimate Yield Strength (MPa)	9.05 ± 0.46	6.91 ± 2.28	2.27 ± 0.53

### Characterization for tensile shape memory properties

**Figure 8:**
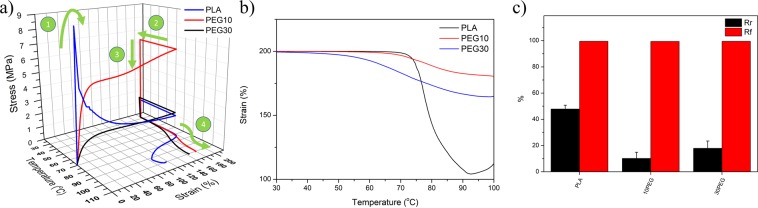
(**a**) 3D shape memory cycle of different PLA/PEG composites: (1) tensile elongation at 70 °C, (2) cooling back to room temperature while maintaining stress, (3) stress release, and (4) reheating and shape recovery; (**b**) recovery behaviour of step 4; (**c**) shape recovery ratio (Rr) and shape fixing ratio (Rf) of different PLA/PEG composites.

Zhang *et al*. provided one of the earliest reports of the shape memory property of PLA^60^. The group theorized that PLA should possess SME, but it was not be observed for PLA due to high brittleness under room temperature conditions. To avoid brittle failure, the team suggested that an elastomer matrix, such as polyamide elastomer (PAE), could be used to enhance the ductility and shape recovery performance. As a result, PLA/elastomer blends became one of the most popular choices for creating a polymeric blend SMP^77,78^. The fabricated composite samples withstood superior tensile strain by up to 500% in the 70 °C environment (Fig. 7). Furthermore, the high stretchability allowed for the composites to withstand the shape memory cycles tests (Fig. 8a). To characterize the shape memory properties, two performance parameters, shape recovery ratio (Rr) and shape fixing ratio (Rf), are defined by the following two equations:

1 $${R}_{r}=\frac{{\varepsilon }_{m}-{\varepsilon }_{p}}{{\varepsilon }_{m}}$$

2 $${R}_{f}=\frac{{\varepsilon }_{u}}{{\varepsilon }_{m}}$$

where *ε*_*m*_ is the applied strain, *ε*_*p*_ is the strain after the recovery, and *ε*_*u*_ is the strain after cooling and shape-fixing.

Samples were first stretched to 200% strain in a 70 °C environment. Afterward, the temperature was lowered back to room temperature and slowly heated to 100 °C to observe recovery behaviour. The high temperature stress-strain curves showed similar dynamics as the results in Fig. 7. A sharp ultimate tensile stress peak was present for pure PLA, while 10PEG plateaued at a higher stress value compared to the 30PEG sample.

All samples achieved high shape fixity properties (close to 100%) as no elastomer matrix was present in the matrix (Fig. 8a). This was responsible for the relatively lower recovery ratio. The unmodified PLA achieved a maximum Rr value close to 50%, which was similar to the findings reported by^77^. It is known that the elastomer matrix is responsible for the recovery process in a polymeric blend SMP by providing elastic recovery at high temperatures^77,79^. Without the elastomer, the crystalline phase in PLA simply softens, and only partial recovery can be observed in the amorphous PLA phase. According to the results of the thermal analysis, the plasticizer lowered the T_g_ of the PLA, which suggested that a decrease in initial recovery temperature can be observed. Additionally, the initial recovery temperature of PLA was higher than 70 °C (Fig. 8b). With 10% PEG, the temperature dropped to around 60 °C, while the 30% PEG sample had a low activation temperature below 40 °C because the plasticizing effect was initiated by PEG.

Despite the fact that actuation can be trigged at a lower temperature by the plasticizer, the plasticizer had a negative influence on the recovery ratio (Fig. 8b,c. An increasing trend in ΔH_m_ was detected by DSC after the incorporation of PEG. Due to the lower interfacial energy at the PEG/PLA region, more PLA crystal nucleation sites were available. The increase in nucleation sites allowed for the PLA crystals to be uniformly distributed within the matrix, which may have separated the amorphous regions in the PLA. These amorphous regions were critical for shape recovery to take place. Therefore, a decrease in the recovery ratio can be expected.

Interestingly, by further increasing the PEG content from 10 to 30 wt%, an improvement in shape recovery can be observed. Such behaviour may be explained by the re-distribution of the amorphous PLA phase due to the excess PEG. In order to achieve the best shape memory performance, it is critical that an optimized crystalline/amorphous phase ratio and distribution is established. With the incorporation of PEG, PLA crystals are able to nucleate at the PEG/PLA interfaces. As a result, an increase in ΔH_m_ can be expected, as shown in Table 1. It should also be noted that the melting enthalpies of the 10PEG and 30PEG are very close to each other, which indicates that the PLA crystal content within the 10PEG is almost saturated and further increasing the PEG will not result in further PLA crystal formation. As a result, the addition PEG will only affect the PLA crystal distribution. In the 10PEG sample, the introduction of PEG separated the amorphous regions that are responsible for the shape recovery, thus a decrease in shape recovery performance can be observed. By further increasing the PEG to 30 wt% the amorphous PLA phases are merged back together, thus a slight improvement in shape recovery was observed.”

### Plasticized 4D materials and their recovery behaviours

**Figure 9:**
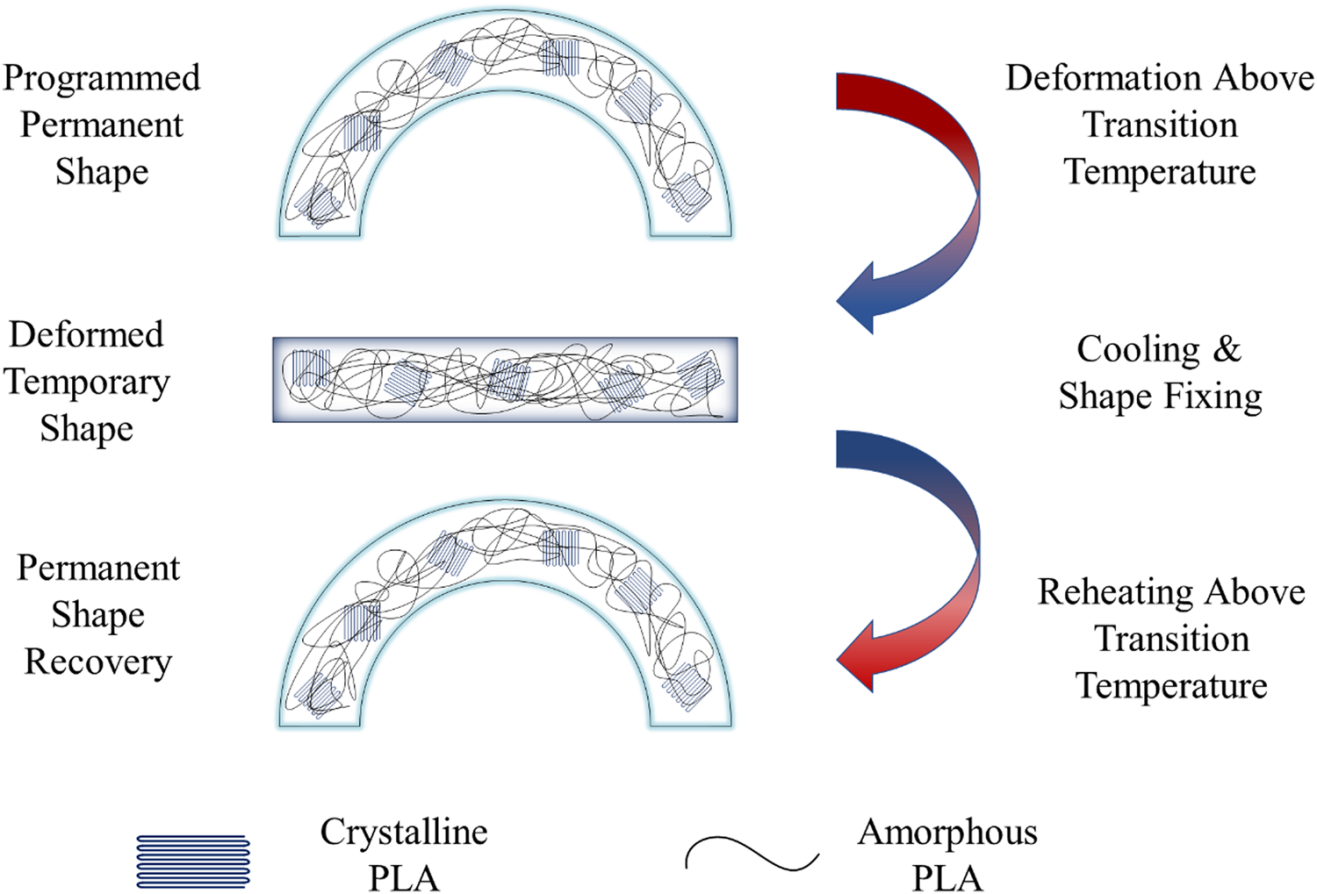
Schematic of shape memory mechanism of arc-like 4D printed PLA sample.

**Figure 10:**
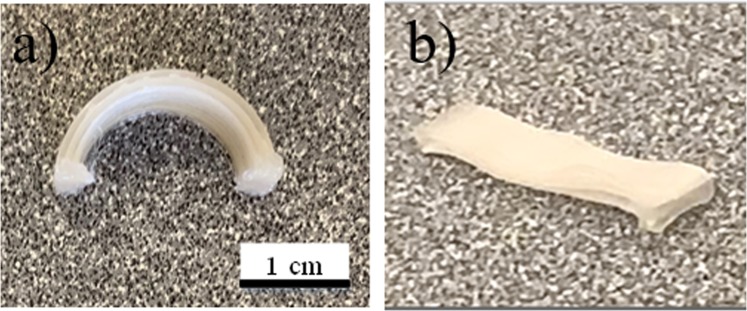
(**a**) 4D-printed PLA arc and (**b**) flattened temporary shape.

To demonstrate the shape memory behaviour, arc samples are designed. The shape memory mechanism is shown Fig. 9. An arc-shape configuration was printed to demonstrate the 4D thermal recovery ability of the fabricated sample (Fig. 10a). Detailed dimensions of the printed model can be found in the Supplementary Materials. The samples needed to be programmed into a temporary shape before 4D recovery could take place. This was done by compressing the arc-like component at 50 °C to prevent any possible brittle failure. The compressed sample was then cooled back to room temperature prior to removal of the applied stress for shape-fixing. The temporary shape training outcome is presented (Fig. 10b). No visible cracks or delamination was observed after the training process.

**Figure 11:**
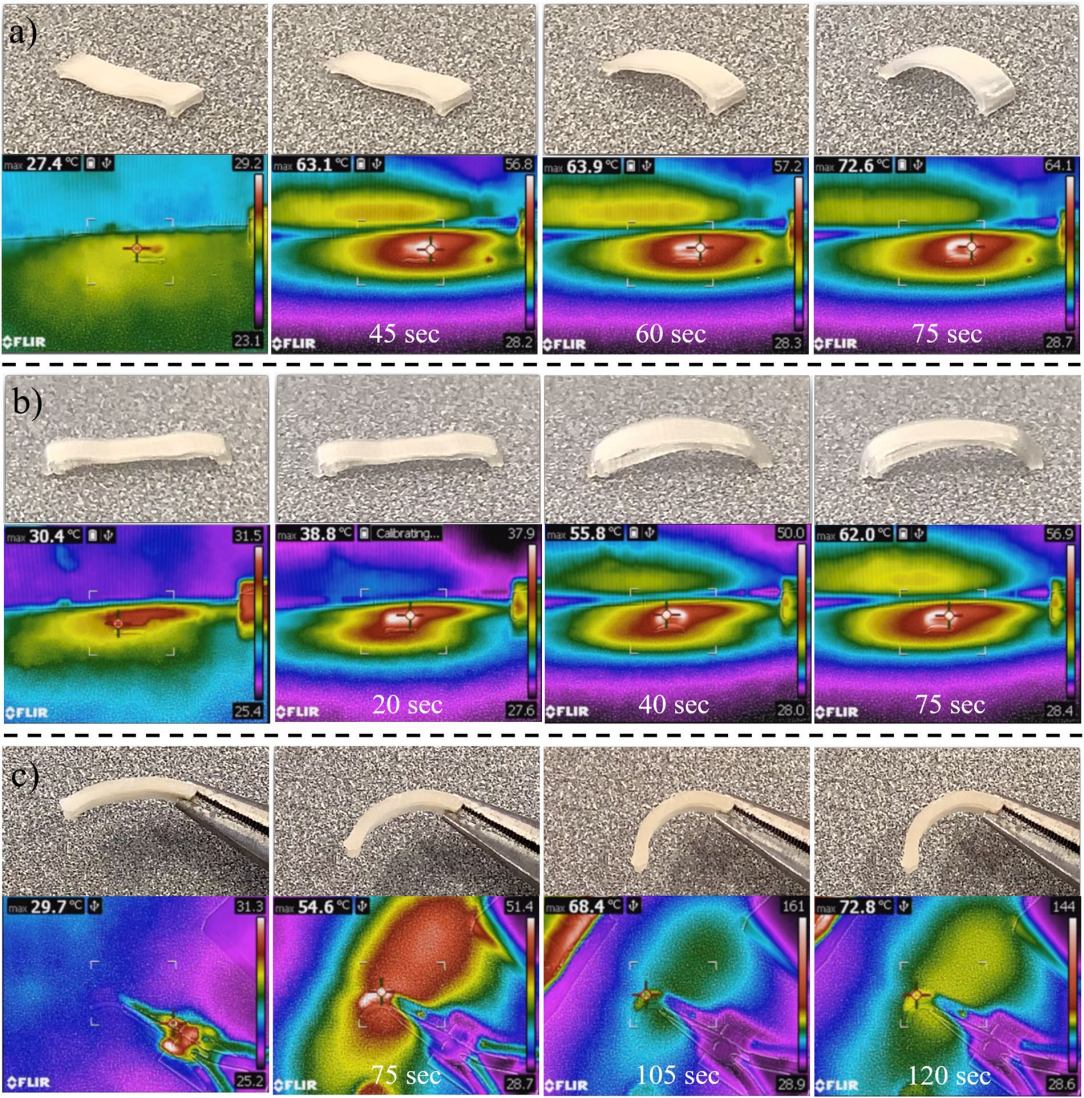
Thermal recovery of 4D-printed (**a**) PLA, (**b**) 10PEG, and (**c**) 30PEG component.

After shape-fixing, a heat gun was utilized above the component to initiate 4D thermal recovery. Videos were taken to visualize thermal recovery, while IR thermal videos were taken to measure the instantaneous temperature. The 4D recovery results for pure and plasticized components are presented (Fig. 11). Complete videos can be found in the Supplementary Materials. All three different compositions recovered to the printed arc-like shape. In addition to the 4D shape memory ability, the plasticizing effect was also observed. The images from the IR camera indicated that the pure PLA sample had an initial recovery temperature above 60 °C, 10PEG had a recovery temperature of 55 °C, and 30PEG had a recovery temperature below 55 °C. This behaviour was anticipated because PEG plasticizer lowered the overall Tg. These results indicate that the actuation temperature of the 4D materials could be fabricated by utilizing a plasticizing effect.

### Functionally graded 4D material with localized recovery ability

**Figure 12:**
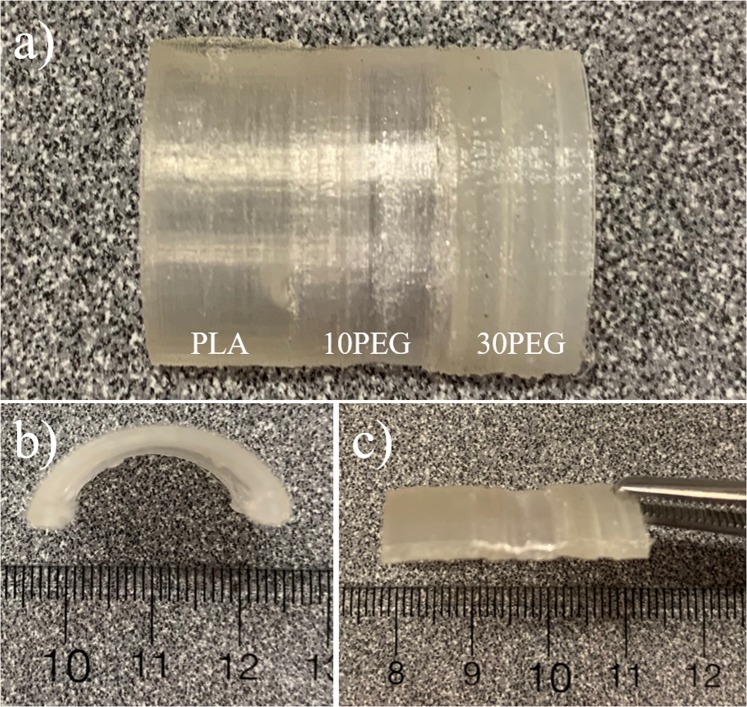
4D-printed functionally graded model.

Since the actuation temperature can be controlled by the amount of plasticizer, it is proposed that a multi-shape actuator can be created by utilizing the property of functionally graded layers. An FG-4D-printed composite fabricated by using a continuous FDM printing technique is shown (Fig. 12). The different weight percent of plasticizer in each layer was visually observable. Similar to the printed arc-like design (Fig. 10), the printed FG-4D model could also be programmed into the flat temporary shape configuration (Fig. 12c) by using the same procedure. The continuous printing process allowed the interfacial layer (PLA/10PEG and 10PEG/30PEG) to have good mechanical properties, and no delamination or cracking was observed at the interfacial region during shape programming.

**Figure 13:**
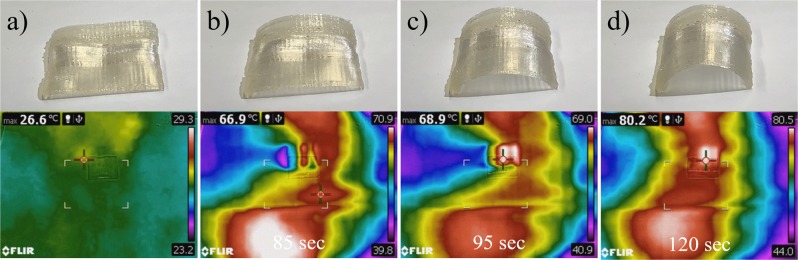
Thermal recovery of 4D functionally graded model.

The thermal recovery of the FG-4D material is presented (Fig. 13). As the temperature rose, the 30PEG layer recovered first and transferred the model into a transitional shape in which one side of the structure was arced while the other side was flat (Fig. 13b). By further increasing the temperature above the Tg of the unmodified PLA, full recovery of the model was achieved (Fig. 13d). A video of this process can be found in the Supplementary Materials. The thermal recovery behaviour observed in this study demonstrated that the transitional shape can be implemented in a 4D-printed structure by utilizing the temperature-dependent properties of the FG plasticized layer, which widens the possible industrial applications of 4D materials.

## Conclusion

In this study, we demonstrated that a 4D-printed material with tunable actuation temperatures can be fabricated using plasticized functionally graded layers. The activation temperature for the shape recovery process can be controlled because the plasticizer content influences the glass transition temperature of PLA. Thermal, viscoelastic, mechanical, and rheological tests indicated that the modified PLA maintains the capacity to be 3D-printed from an FDM printer. Shape memory properties were characterized and showed that the desired 4D recovery behaviour can be achieved. Lastly, the FG-4D material with localized actuation behaviour was successfully fabricated.

## Methods

PLA (4032D, NaturedWorks Inc.) pellets were first dried at 80 °C for 24 hours prior to any treatment. Next, 10 wt% and 30 wt% PEG (2000 average molecular weight, Sigma Aldrich) was used as a plasticizer by melt-blending to PLA using a micro-compounder (DSM Xplore 15) at 170 °C and 100 RPM for 10 minutes. After compounding, the 3D-printable PLA/PEG filaments were fabricated by attaching an automatic roller at the end of the compounder. For material characterization, parts of the composite filaments were cut, transferred to a hot compression press (Carver Model 4386), and remolded at 170 °C under 5 metric tons of pressure. A customized 3D FDM printer (BigBox, BigBox 3D Ltd.) was utilized to fabricate the 4D components using a standard 0.3-mm nozzle with a nozzle temperature of 180 °C. Arc-shaped components were printed, which can be reshaped into a flat configuration once subjected to compression. The geometry of the component can be found in the Supplementary Information. The FG trilayer component was fabricated using a standard printing process segmented into three sections during which extruder motion was paused for filament exchange.

An analysis of the prefabricated composites and the 4D-printed components was conducted. Scanning electron microscopy (SEM, IT-100, JEOL, Inc.) was used to study the plasticizing effect at the microscopic level and the cross-sectional morphology of the 4D FG layers. To verify the presence of PEG, Fourier transform infrared spectroscopy (FTIR, Alpha, Platinum-ATR, Bruker, Inc.) was conducted. Differential scanning calorimetry (DSC, Q2000, TA Instrument) was used to study the glass transition and melting behaviour, and thermogravimetric analysis (TGA, Q50, TA Instrument) was performed to characterize the degradation of the composites. During DSC testing, samples were heated from –50 °C to 230 °C at a heating rate of 10 °C/min. For TGA, samples were heated from 30oC to 600 °C at a heating rate of 20 °C/min under a nitrogen atmosphere. A dynamic mechanical analyzer (DMA, Q800, TA Instrument) was used to characterize the mechanical, viscoelastic, and shape memory properties. Frequency sweep tests were conducted at room temperature with a 15-μm oscillation from 1 Hz to 50 Hz. For temperature ramp experiments, samples were first subjected to isothermal condition at 35 °C for 5 minutes, which was later increased to 100 °C at a heating rate of 3 °C/min under a constant 15-μm oscillation at 1 Hz. To study the high-temperature viscosity and plasticizing effects, rheological measurements (ARES, TA Instrument) were conducted. A parallel-plate rotational fixture was utilized while tests were conducted at 190 °C at an oscillation of 1 Hz to 100 Hz under a constant 1% oscillatory strain. Mechanical testing was conducted at both room temperature and a high temperature (70 °C). Due to the high stiffness behaviour of PLA, tensile tests at room temperature were conducted using a mechanical tensile tester (Instron 5848 MicroTester, Instron), while the DMA was used for high-temperature testing. Due to the machine constraint, room temperature tensile testing samples have a geometry of 10 mm in length, 7 mm in width, and 0.4 mm in thickness while DMA testing sample have a geometry of 5 mm in length, 4 mm in width, and 0.2 mm in thickness. For the tensile testing, a 5 mm/min extension was applied until failure. For DMA testing, samples were first conditioned at an isothermal testing temperature (70 °C) prior to applying a 50% strain/min rate until failure. To verify the SME, samples were conditioned at 70 °C and stretched to 200% strain with 50% strain/min rate. Both stress and strain were fixed as the temperature was lowered to room temperature. With stress removed, a heating rate of 3 °C/min was applied, and the amount of strain recovery was recorded.

To demonstrate 4D recovery, 3D-printed components were first deformed at 50 °C into a flat configuration. For the recovery process, a heat gun was placed directly above the flattened sample while the increase in temperature was simultaneously measured using an infrared radiation (IR) thermal camera (C3, FLIR).

## Supplementary information

SUPPLEMENTARY

4D FG x8 speed

4D FG thermo x8 speed

10PEG x4 speed

10PEG thermo x4 speed

30PEG x8 speed

30PEG thermo x8 speed

PLA x4 speed

PLA thermo x4 speed
