# Supplementary material for: 4D-printed hybrids with localized shape memory behaviour: Implementation in a functionally graded structure
Source: Sci Rep. 2019 Dec 10;9:18754. doi: 10.1038/s41598-019-55298-1 (PMC6904723; doi:10.1038/s41598-019-55298-1)
Supplement: Supplementary file 1 — SUPPLEMENTARY [file 41598_2019_55298_MOESM1_ESM.pdf]

TITLE: 4D printed hybrids with localized shape memory behaviour: implementation in functionally graded structure

Yu-Chen Sun, Yimei Wan, Ryan Nam, Marco Chu and Hani E. Naguib*

Department of Mechanical and Industrial Engineering, University of Toronto, Toronto, Canada

Department of Materials Science and Engineering, University of Toronto, Toronto, Canada

Institute of Biomaterials and Biomedical Engineering, University of Toronto, Toronto, Canada

5 Kings College Rd., Toronto, Ontario, Canada, M5S3G8

Contact email: naguib@mie.utoronto.ca

Supplementary Document

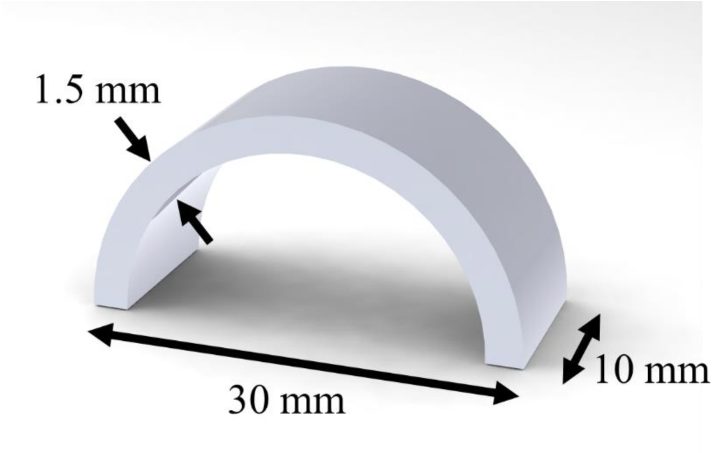

Figure S1 *– Geometry of 3D printed arc-shape component*
